# Work performance of middle-aged and elderly employees in hotel industry: the moderating effects of organizational support and age discrimination

**DOI:** 10.3389/fpsyg.2025.1377368

**Published:** 2025-04-28

**Authors:** Wen Wei Chow, Pai Peng

**Affiliations:** ^1^School of Management, Xiamen University, Xiamen, China; ^2^School of Geography and Ocean Science, Nanjing University, Nanjing, China

**Keywords:** middle-aged and elderly employee, three-dimensional capital, organization support, age discrimination, hotel industry

## Abstract

With the acceleration of the process of social aging, the re-employment of middle-aged and elderly people has gradually attracted the attention of society. Due to the relatively low barrier to entry, the hotel industry is favored by middle-aged and elderly people who are re-employed. This study analyzes the impacts of the three-dimensional capital on the job performance of middle-aged and elderly employees and investigates how organizational support and age discrimination moderate the effect of job satisfaction on job performance. A self-administered questionnaire was distributed to 400 hotel employees using convenience sampling. First, the three-dimensional capital has a positive impact on the job performance of both middle-aged and elderly employees; second, Job satisfaction has a mediating effect between the three-dimensional capital and the job performance of middle-aged and elderly employees; third, perceived organizational support positively moderates the effect of job satisfaction on job performance of middle-aged and elderly employees, and age discrimination perception negatively moderates the effect of job satisfaction on job performance of middle-aged and elderly employees. Theoretical and practical implications, as well as limitations and future research directions, are discussed.

## 1 Introduction

With the increase in life expectancy and the decrease in the fertility rate, and other factors, the phenomenon of population aging has become more and more common worldwide. More developing countries have also become an aging society, population aging is increasingly becoming a global concern. According to recent projections by the United Nations Department of Economic and Social Affairs (2022), the proportion of the global population aged 65 years or over worldwide will increase from 9.7% in 2022 to 16.4% in 2050. The report indicates that, by 2050, at least one in four will be aged 65 years or older in regions including Europe and Northern America, and Eastern and South-Eastern Asia. The elderly population generally relies on support from their children and social security. As the aging process accelerates, older individuals increasingly become an economic burden on both society and families. Consequently, promoting the employment of middle-aged and elderly individuals and actively utilizing their labor force has become a key strategy to mitigate the challenges of population aging (Chen and Li, 2024). The re-employment of middle-aged and elderly individuals is particularly significant in reducing the social security burden and fostering sustainable social development.

Due to the aging of the population, the life cycle is also remodeled from the simple three stages of the childhood-working period-retirement period to four stages of childhood-middle age-active old age-old age. The middle-aged and the elderly as those from 55 to 64 (Flynn et al., 2013), the aging of the population will greatly change the demographic structure of a country, and it will also have a massive impact on all walks of life. Present studies showed that age is significantly and negatively associated with work ability (Sottimano et al., 2019). However, the early legal retirement age, rapidly changing labor demand, family care burden, and unfriendly labor market system have combined to prevent the retired population from re-entering the labor market, making the labor force participation rate of the retired population consistently low (Cheng and Li, 2022). Meanwhile, more and more aging employers mean a shortage of working-age laborers. This also makes companies consider hiring older people for employment more than they did in the past. This study aims to explore the differences in middle-aged and elderly and non-middle-aged and elderly employees’ job performance in the hotel industry from the perspective of three-dimensional capital. It also tends to introduce age discrimination perception as a regulatory variable to analyze this difference.

The study makes significant contributions to the existing body of knowledge. First, this research enriches the existing research by investigating the effects of three-dimensional capital on middle-aged and older employees’ performance in the hotel industry. Compared with the findings of Huang et al., we further compared the differences in middle-aged and older employees’ and non-middle-aged employees’ job performance, making the research conclusions richer (Huang et al., 2021). Second, we identified job satisfaction as the mediating role between three-dimensional capital and employees’ job performance. Previous studies have directly examined the effect of three-dimensional capital but ignored the link between three-dimensional capital and the outcome variable (Zientara et al., 2023), we investigated the mediating effects of job satisfaction. Third, we contribute to the literature on job performance by identifying two new moderating variables, including perceived organizational support and age discrimination perception. Our study provides empirical evidence that the perceived organizational support and age discrimination perception moderate the relationship between job satisfaction and job performance.

## 2 Literature review and hypotheses development

Recent studies have increasingly focused on the role of middle-aged and elderly employees in the workforce, particularly in the context of an aging global population. Research by Karami et al. (2017) highlights the importance of social and psychological capital in enhancing job performance for older workers, they found that self-efficacy, resilience, and social support are significant predictors of job satisfaction among middle-aged and elderly employees. Research by Wissemann et al. (2022) emphasizes the importance of strategic guidance and technological solutions in human resources management to sustain an aging workforce. Such research underscores the need for diverse and interdisciplinary approaches to support aging employees, reflecting varying regional strategies to promote their inclusion in the labor market.

Existing research often examines individual components of capital, overlooking their combined effects and the interaction between them, particularly in specific industries like hospitality. This study fills this gap by analyzing the collective impact of three-dimensional capital on job performance among middle-aged and elderly employees in the hotel industry. Additionally, while previous studies have focused on the direct effects of these capitals, the role of job satisfaction as a mediator has not been fully explored. This study clarifies how job satisfaction influences the relationship between three-dimensional capital and job performance. Finally, the moderating effects of age discrimination perception and perceived organizational support on the job satisfaction-job performance link have been underexplored. This research sheds light on how these factors impact older employees’ job outcomes, offering insights for more targeted management strategies.

### 2.1 Three-dimensional capital of middle-aged and elderly employees

Capital is a key resource in parallel with land, labor, and technology that constitute the basic economic production system and contribute to economic development and growth. Previous studies suggested that human knowledge and ability, such as education, experience, and skills, are also a form of capital that can contribute to the effectiveness and performance of an organization (Hsu et al., 2007). Huang et al. (2021) identify three types of capital based on previous studies, including human capital, social capital, and psychological capital. Human capital posits that people can gain a form of capital through education, schooling, and training, which then provides an internal capability or resource (Chowdhury et al., 2014). Social capital is built upon people’s social connections, mutual obligations and expectations, reciprocity, and social norms. In addition, social capital is considered to comprise three salient dimensions, including structural social capital, relational social capital, and cognitive social capital (Li et al., 2021). Psychological capital is defined as an individual’s positive psychological state and is reflected in four aspects: self-efficacy, optimism, hope, and resilience (Zhou et al., 2022).

Previous studies in seven 5-star hotels in China found that psychological capital is the strongest predictor of employees’ self-reported job performance. In addition, education and work experience, as components of human capital, influenced both self-reported and supervisor-rated job performance (Huang et al., 2021). Three-dimensional capital primarily highlights the unique advantages of elderly employees, such as their accumulated human capital, relatively low investment costs, and the potential benefits these offer for hotel staff development. The availability of aged labor resources can play a key role in ensuring labor supply during China’s transition from “deindustrialization” to “reindustrialization.” Middle-aged and elderly employees have incomparable advantages in terms of human capital, social capital, and psychological capital compared with other age groups. Specifically, their enriched work experience and working skills reflect the human capital. The excellent social resources and network relationships suggested the social capital advantages. The human resources of the elderly have advantages that other age groups do not have: knowledge capital advantage, network capital or relationship capital advantage, relatively low cost, fast and high yield of human capital investment, and readiness to use. In general, middle-aged employees nowadays have a higher level of human capital, work experience, and computer skills than middle-aged employees of previous generations; they may even be at a level that competes with young people entering the market now (Sinclair et al., 2024).

### 2.2 Three-dimensional capital and job performance

Human capital refers to the knowledge, experience, skills, and health that employees acquire through education, migration, practical experience, health care, training, and others (Schultz, 1960). Alkharafi (2024) believe that human capital, social capital, and psychological capital have an essential impact on migrant workers returning to their hometowns to start businesses. However, there are certain shortcomings in its current capital situation, lack of human capital affecting the motivation of their entrepreneurial willingness, weak social capital affecting the actual performance of their entrepreneurship, and lack of psychological capital affecting their entrepreneurial performance. Therefore, it is necessary to find the correct promotion strategy in the three aspects of human capital, social capital, and psychological capital.”

Taylor, the father of scientific management, made scientific choices for workers and trained them to use standard operating methods; thus, they were allowed to make progress, improving production efficiency. This experiment shows the importance of improving human capital to corporate performance. Years of education, accumulated work skills, and rich professional knowledge of employees can promote the growth of corporate performance. Ke et al. (2010) examined the relationship between the human capital of executives and employees and corporate performance through a survey of 197 listed companies. The research shows that both the human capital of executives and employees will bring apparent direct effects on corporate performance. Tian and Xie (2012) used the utility analysis to find that employees’ human capital is positively related to their performance through two types of field surveys of 363 samples. Based on it, this research proposes the following assumption:

*H1a*: Middle-aged and elderly employees’ human capital has a positive impact on job performance.

Social capital refers to the network of employees obtained through various social relationships, as well as the relationship-related resources that contribute to the advantage corporations gain in the process of development (Portes, 1998). To finish a task, employees need to communicate and cooperate reasonably with colleagues. Rich social capital helps individuals to obtain external support. Based on the theory of social exchange and social norms, individuals with rich social capital will be more willing to help others and have higher work enthusiasm and dedication to gratitude others for help and maintain social networks in the meantime. Ke et al. (2010) have obtained empirical research and found that the individual’s social capital significantly impacts employees’ job performance. Adler and Kwon (2002) found that social capital significantly impacts employees’ career choices, salary, and performance. Based on it, we propose the following assumption:

*H1b*: Middle-aged and elderly employees’ social capital has a positive impact on job performance.

Psychological capital refers to employees’ positive psychology in learning and self-improvement (Luthans and Youssef, 2004). It surpasses other capital and belongs to core factors. Nowadays, rapid technological, economic, and social changes have brought psychological problems like stress and anxiety to workers (Li et al., 2018). Healthy psychology becomes more and more critical for employees. According to an empirical study of 198 pairs of direct leaders and employees, Zhong (2007) proposed that employees’ hope, optimism, and tenacity positively impact their job performance. Through an empirical investigation of engineers. Based on this, we propose the following assumption:

*H1c*: Middle-aged and elderly employees’ psychological capital has a positive impact on job performance.

### 2.3 Mediating effect of job satisfaction

Job satisfaction is the positive mental state composed of satisfaction about every aspect of the job. Employees with higher job satisfaction are more motivated to work and have higher job performance. Job satisfaction is a crucial attitude and will significantly impact employees’ job performance and behavior (Berhanu, 2023). Based on the motivation theory based on expectations. Schleicher et al. believe that job satisfaction and job performance are mutual cause and effect; job satisfaction can affect job performance and can be improved through job performance. This model is widely accepted due to its integrity (Schleicher et al., 2004). Job satisfaction is a well-established factor influencing employee behavior, performance, and overall well-being (Cao et al., 2022). It encompasses various aspects of the work experience, including employees’ perceptions of their work environment, roles, and the support they receive. Job satisfaction is shaped by multiple factors, including human capital (skills and experience), social capital (relationships and support networks), and psychological capital (self-esteem, optimism, and resilience). By conceptualizing job satisfaction as a mediator, this study explores not only the direct impact of these different forms of capital but also how they interact with employees’ emotional and cognitive responses to their work. This interaction ultimately influences their job performance, offering a deeper understanding of how capital affects employee outcomes.

Corporates’ investment in human capital can increase the organizational commitment of employees to a certain extent and improve job satisfaction. Yen et al. pointed out that social capital affects the job satisfaction of employees (Yen et al., 2020). Employees’ hope, optimism, and resilience have a positive relationship with their job performance, job satisfaction, and happiness (Aggarwal, 2024). Furthermore, the integrated psychological capital effect is more significant than any single dimension (Abbas et al., 2022). In summary, this research proposes hypotheses as follows:

*H2a*: Job satisfaction mediates the effect of middle-aged and elderly employees’ human capital on job performance.

*H2b*: Job satisfaction mediates the effect of middle-aged and elderly employees’ social capital on job performance.

*H2c*: Job satisfaction mediates the effect of middle-aged and elderly employees’ psychological capital on job performance.

### 2.4 Moderating effect of perceived organizational support

Perceived organizational support refers to employees’ perceptions and opinions about how employers judge their corporate contributions and how employers care about their welfare. According to the theory of Social Exchange, the stable relationship between employees and organizations is based on the balance between the supply and demand of both parties; good perceived organizational support can help improve employees’ attitudes and behaviors (Bui Thi and Mai, 2024). When the organization emphasizes the responsibilities and obligations of employees, it will help to maintain the “supply–demand balance” between the organization and internal customers (Bai et al., 2023). Moreover, it encourages employees to spontaneously take positive attitude and behavior toward the job if it can respond to and meet the various work and psychological needs of employees simultaneously (Chillakuri and Vanka, 2022).

Wang et al. investigated the influence of organizational sustainability performance from the perspective of organizational support as perceived by employees. Results show that perceived organizational support has a positive and significant effect on organizational sustainability performance (Wang et al., 2018). Through a study of workers, Chong et al. proposed that employees with a high perceived organizational support have a more positive attitude toward just-in-time (JIT); their work performance has improved significantly (Chong et al., 2001). The research of Bell and Menguc (2002) on salespeople shows that employees with good perceived organizational support have relatively high customer evaluations of service quality. Therefore, this research proposes the hypothesis:

*H3*: Middle-aged and elderly employees’ perceived organizational support positively moderates the impact of job satisfaction on job performance.

### 2.5 Moderating effect of age discrimination perception

Age discrimination refers to the negative judgment or unreasonable treatment of others due to their age (older or younger). This phenomenon is common in enterprises. Age discrimination perception originates from the discussion of aging (Berger, 2004). Furthermore, with the development of society, age discrimination appears in different industries. Based on the theory of achievement demand, achievement need refers to the need of individuals to work hard to obtain success and gain a sense of superiority. When employees realize age discrimination, they will feel alienated from the organization, reduce job satisfaction, and affect job performance (Hooker et al., 2019).

**Figure 1:**
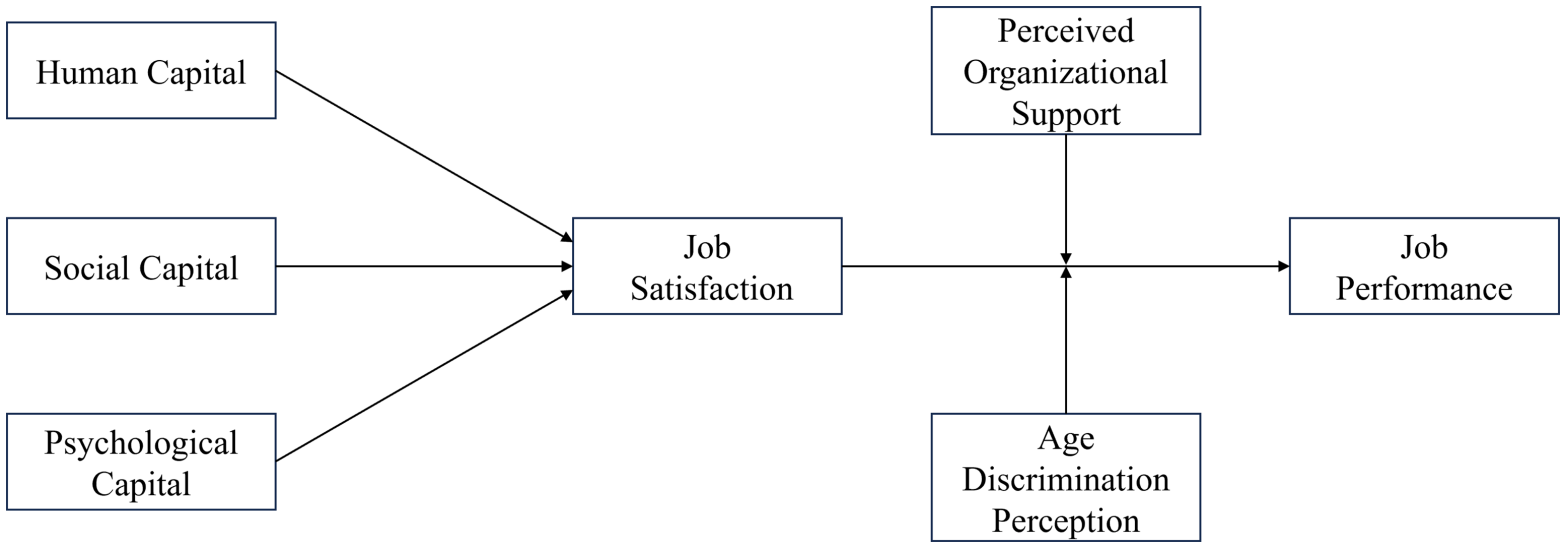
Conceptual model and hypothesized relationships.

Zhang and Wei believe that age discrimination perception will impact employees’ work-shrinking behavior (Zhang and Wei, 2018). The stronger the employee’s sense of age discrimination perception, the more pronounced work withdrawal behavior is. In other words, employees would reduce their working time and avoid taking more responsibility. Griffin et al. (2016) presented results from two 3-wave longitudinal studies of differing time lags demonstrating the direct negative effect of perceived discrimination directed at middle-aged and elderly employees on both job satisfaction and actual job withdrawal but not on actual retirement. Therefore, this research proposes the hypothesis:

*H4*: Middle-aged and elderly employees’ age discrimination perception negatively moderates the impact of job satisfaction on job performance (Figure 1).

## 3 Methodology

### 3.1 Pilot test

The rapid population aging is considered one of the most prominent social and economic challenges facing China. Therefore, the population of this study consists of elderly employees of hotels in Taiwan, China. In order to ensure the reliability and validity of the scale, this study pre-tests some employees in a hotel. This paper pre-tests the subjects from November 11 to November 21, 2022, to ensure their resonance. The author distributed 80 questionnaires and returned 76 valid questionnaires. According to the results of the questionnaire collection, the reliability and validity test is carried out to correct the scale. Formal questionnaires are conducted after removing items not discriminating and with low validity.

### 3.2 Instrument development

The questionnaire used in this study included five sections. Constructs employed in this study were operationalized using multi-item scales from past studies. The measurement of human capital refers to the three items that Ke et al. used to measure employees’ human capital. Moreover, the item of “health” is added according to the elements contained in human capital. The measurement of social capital contains three aspects: colleague relationship, leadership relationship, and external relationship (Ke et al., 2010). According to Luthans and Jensen, psychological capital refers to individuals’ positive attitudes during their growth and development, measured by the psychological capital scale (PCQ). The measurement of job satisfaction is based on the questionnaire developed by Yang et al. (2010). The questionnaire is adapted from the scales developed by scholars at home and abroad, especially the Minnesota Satisfaction Short Scale (MSQ) developed by Weiss et al. (1967). There are 12 items, three dimensions that include the job itself, the interpersonal relationship, and salary. The measurement of job performance has used the scale revised by Wang et al., which includes four dimensions: task performance, interpersonal facilitation, and job dedication (Wang et al., 2004). The job dedication and interpersonal facilitation each include five items, while the job dedication includes six items, for a total of 16 items. Studies have verified the applicability of the scale in the Chinese cultural context, and the scale has good reliability and validity. This study uses a short scale developed by Eisenberger et al. (1997), which includes two dimensions (job support and social–emotional support), with eight items in total to measure perceived organizational support. Adopted the scale developed by Marchiondo et al. (2016), the study measures age discrimination perception from the employees’ perspective and gained a scale that is suitable for young, middle-aged, and elderly employees. The scale consists of 9 items, such as “Because of my age, my work role or task is ignored,” and “My contribution is not valued because of my age.”

### 3.3 Data analysis

This study employed AMOS and SPSS statistical software to analyze the collected data. Exploratory factor analysis using the Harmon test confirmed the absence of Common Method Variance (CMV) issues. Confirmatory factor analysis, reliability analysis, Composite Construct Reliability (CCR), and Average Variance Extracted (AVE) were conducted to assess the reliability and validity of the measurement items. Correlation analysis was employed to examine the relationship between the items and research hypotheses, and structural equation modeling and multi-group analysis were conducted to test the hypotheses. To ensure the validity and reliability of the research findings, a series of measures were taken during data collection and analysis to minimize the impact of biases and unobserved variables. Multiple potential confounding variables were controlled for during the analysis, and sensitivity analysis was conducted to assess the robustness of the results. In addition, the sample selection and measurement tools used in the study were rigorously validated to ensure the credibility of the research findings.

## 4 Results

### 4.1 Descriptive statistics analysis

The survey in this research is divided into two questionnaires. The first questionnaire is the self-evaluation survey conducted by employees, and the second questionnaire is for employee supervisors who score employee job performance. In order to obtain the sample size used in this research, the author conducted a questionnaire survey in 7 three to five-star hotels’ overall operations including front and back offices in Taiwan from March 16, 2023, to April 30, 2023. Five hundred sixty-five questionnaires were issued, 432 were returned, and 400 were valid questionnaires; the effective sample recovery rate is 71%. Among the effective samples, middle-aged and elderly samples are 168, and the number of non-middle-aged and elderly samples is 232 (Table 1).

**Table 1:** Profile of the sample (*n* = 400).

Variables	Items	*N*	Percentage
Gender	Men	183	46%
	Women	217	54%
Age	Middle-aged and elderly	168	42%
	Non-middle-aged and elderly	232	58%
Education	Senior high school and below	109	27%
	College	176	44%
	Undergraduate	90	23%
	Graduate and above	25	6%
Hotel Star	Three-star and below	151	38%
	Four-star	162	41%
	Five-star	87	22%

### 4.2 Measurement model

This research conducted a reliability test on all items and obtained Cranach’s results as shown in Table 2. The reliability coefficients of each variable are greater than 0.7, indicating that the questionnaire reliability is good.

**Table 2:** Reliability coefficients of various variables.

	Cranach’s α	KMO	Barlett	Total variance explained	df	*p*
Human capital	0.854	0.821	673.053	69.50%	6	0.000
Social capital	0.823	0.72	430.156	73.91%	3	0.000
Psychological capital	0.973	0.988	7529.02	62.02%	276	0.000
Satisfaction of job	0.869	0.828	755.075	71.85%	6	0.000
Satisfaction of interpersonal relationship	0.864	0.826	724.779	71.03%	6	0.000
Satisfaction of salary	0.86	0.828	702.592	70.51%	6	0.000
Job support	0.878	0.838	803.37	73.25%	6	0.000
Social–emotional support	0.88	0.834	819.903	73.60%	6	0.000
Age discrimination perception	0.939	0.961	2469.606	67.15%	36	0.000
Job dedication	0.894	0.882	1084.274	70.22%	10	0.000
Task performance	0.916	0.924	1485.389	70.34%	15	0.000
Interpersonal facilitation	0.896	0.888	1102.837	70.75%	10	0.000

Research has shown that factors have higher loadings, indicating a higher degree of variability that can be explained by potential factors. The main fitting indices for structural model testing are shown in the table, where χ2/df = 1.073(<3.0), RMSER = 0.014(<0.08), IFI = 0.991(>0.9), CFI = 0.991(>0.9), PGFI = 0.798(>0.5), PNFI = 0.854(>0.5), PCFI = 0.911(>0.5). The fitting indices of the obtained statistical test quantities are all within the range of the adaptation criteria, indicating that the overall structure of this theoretical model is reasonable, with good adaptability and good structural validity.

With AMOS and SPSS software, the maximum likelihood estimation method was used to perform confirmatory factor analysis and composite reliability test to obtain the average variance extraction (AVE) of each variable: human capital 0.572, psychological capital 0.587, social capital 0.592, job satisfaction 0.601, job performance 0.614, perceived organizational support 0.649, age discrimination perception 0.631. The above AVE values are all greater than 0.5, which confirms that the six variables related to this study have excellent convergent validity (Table 3).

**Table 3:** AVE value and CR value of each variable.

Variable	Average variance extraction value (AVE)	Composite reliability value (CR)
Human capital	0.572	0.842
Social capital	0.592	0.813
Psychological capital	0.587	0.972
Job satisfaction	0.601	0.948
Job performance	0.614	0.962
Perceived organizational support	0.649	0.937
Age discrimination perception	0.631	0.939

By comparing the correlation coefficient between the variables and the average variance extraction of the variables, it is found that the square root of the average variance extraction of each variable itself is more significant than its correlation coefficient with other variables, indicating that the variables in this scale have higher discriminant validity (Table 4).

**Table 4:** Discriminant validity test of each variable.

Variables	HC	SC	PC	JS	POS	ADP	JP
HC	0.756						
SC	0.399**	0.769					
PC	0.399**	0.502**	0.766				
JS	0.445**	0.428**	0.357**	0.775			
POS	0.187**	0.248**	0.172**	0.545**	0.806		
ADP	−0.176**	−0.212**	−0.164**	−0.476**	−0.643**	0.794	
JP	0.473**	0.428**	0.373**	0.441**	0.199**	−0.172**	0.784

HC = Human Capital, SC = Social Capital, PC = Psychological Capital, JS = Job Satisfaction, JP = Job Performance, POS = Perceived Organizational Support, ADP = Age Discrimination Perception. The diagonal elements are the squared root of the average variance extracted; the off-diagonal elements are the correlations among the constructs. Correlation: *p < 0.05, **p < 0. 01, ***p < 0.001.

### 4.3 Results of main and mediating effect testing

To examine whether there was a severe issue of multicollinearity among variables, this study conducted a variance inflation factor (VIF) test before hypothesis testing. The results indicated that the VIF values of all the models ranged from 1.403 to 1.961, all of which were less than 5, indicating the absence of multicollinearity issues. This study employed hierarchical regression analysis using SPSS to test the research hypotheses. The results are presented in Table 5. Controlling for covariates (gender, age, education, and hotel star), this study first tested H1a, H1b, and H1c, and found that human capital, social capital, and psychological capital have a significant positive impact on work performance (M2, *β* = 0.201, *p* < 0.001; M3, β = 0.214, *p* < 0.001; M4, β = 0.348, p < 0.001), supporting the acceptance of H1a, H1b, and H1c.

**Table 5:** Results of three-dimensional capital on job performance.

Variable	M1	M2	M3	M4
Human capital		0.201***		
Social capital			0.214***	
Psychological capital				0.348***
Gender	−0.005	0.005	−0.014	0.007
Age	0.673***	0.587***	0.602***	0.625***
Education level	0.131***	0.110***	0.117***	0.117***
Hotel star	−0.014	0.001	0.012	−0.001
*R* ^2^	0.486***	0.518***	0.526***	0.505***
Adjusted *R*^2^	0.481***	0.512***	0.520***	0.499***
*F*	93.457***	84.781***	87.421***	80.410***

Correlation: *p < 0.05, **p < 0.01, ***p < 0.001.

To test the mediator role of job satisfaction in human capital, social capital, psychological capital, and job performance, this research uses the three-dimensional capital of middle-aged and elderly employees as independent variables for job satisfaction and job performance. It also utilizes job satisfaction as an independent variable to regress job performance, following the steps of the above mediating test. Results are shown in Table 6.

**Table 6:** Mediating testing effect analysis of job satisfaction.

Model	Variable	Non-standardized coefficient		Standardized coefficient	*t*	Sig.	*F*	*R* ^2^	Adjusted *R*^2^
		*β*	SE	*β*					
M5	Constant	1.143	0.156		7.338	0.000			
	HC	0.339	0.046	0.145	7.314	0.000	59.423***	0.290***	0.287***
	JS	0.288	0.047	0.288	6.09	0.000			
M6	Constant	1.249	0.157		7.98	0.000			
	SC	0.276	0.045	0.292	6.137	0.000	71.338***	0.264***	0.261***
	JS	0.316	0.048	0.316	6.633	0.000			
M7	Constant	1.191	0.169		7.038	0.000			
	PC	0.255	0.048	0.247	5.305	0.000	65.433***	0.248***	0.244***
	JS	0.353	0.047	0.353	7.57	0.000			

Correlation: *p < 0.05, **p < 0.01, ***p < 0.001.

According to the regression analysis of the three-dimensional capital of middle-aged and elderly employees on their job performance, the F tests of the nine models are all significant at the level of 0.001. Table 6 shows that when examining the mediator role of job satisfaction in the three-dimensional capital of middle-aged and elderly employees and job performance, the *p-*values of the nine models are all less than 0.05, indicating that the above linear regression models are significant. Since the absolute value of the regression coefficient of human capital on job performance in the mediation model (0.145) is less than the absolute value of the regression coefficient of human capital on job performance (0.201), the result suggests that job satisfaction has a partial mediator role in the relationship between human capital and job performance. It verifies hypothesis H2a. The absolute value of the regression coefficient of social capital on job performance in the mediation model (0.192) is less than the absolute value of the regression coefficient of social capital on job performance (0.214). Therefore, job satisfaction partially mediates the relationship between social capital and job performance, which verifies hypothesis H2b. The absolute value of the regression coefficient of psychological capital on job performance in the mediation model (0.247) is smaller than the absolute value of the regression coefficient of psychological capital on job performance (0.348), so there is a partial mediator role of job satisfaction in the relationship between psychological capital and job performance, which verifies the hypothesis H2c.

### 4.4 Moderating effect testing

#### 4.4.1 Moderating effect of perceived organizational support

Through regression analysis of job satisfaction, perceived organizational support, and job performance, it can be seen that the standardized coefficient of job satisfaction * perceived organizational support (i.e., interactions in Table 7) is 0.268; the *p* value is significant at the level of 0.001; the *F* values of the two models are 65.433 and 38.483; the△R^2^ is significant at the level of 0.05. These numbers indicate that the regression fit is good. There is a positive moderating effect of perceived organizational support between job satisfaction and job performance; hence hypothesis H3 is verified.

**Table 7:** Moderating effect analysis of perceived organizational support.

Model	Variable	Non-standardized coefficient		Standardized coefficient	t	Sig.	*F*	*R* ^2^	Adjusted *R*^2^
		*β*	SE	*β*					
M8	Constant	4.602	0.464		10.31	0.000			
	JS	0.326	0.152	0.301	2.128	0.034	72.731***	0.334***	0.329***
	POS	0.235	0.005	0.212	6.030	0.000			
M9	Constant	3.809	0.555		6.856	0.000			
	JS	−0.251	0.196	−0.25	−1.278	0.202	38.483***	0.226***	0.220***
	POS	−0.724	0.181	−0.765	−3.991	0.000			
	JS*POS	0.223	0.058	0.268	3.833	0.000			

Correlation: *p < 0.05, **p < 0.01, ***p < 0.001.

**Figure 2:**
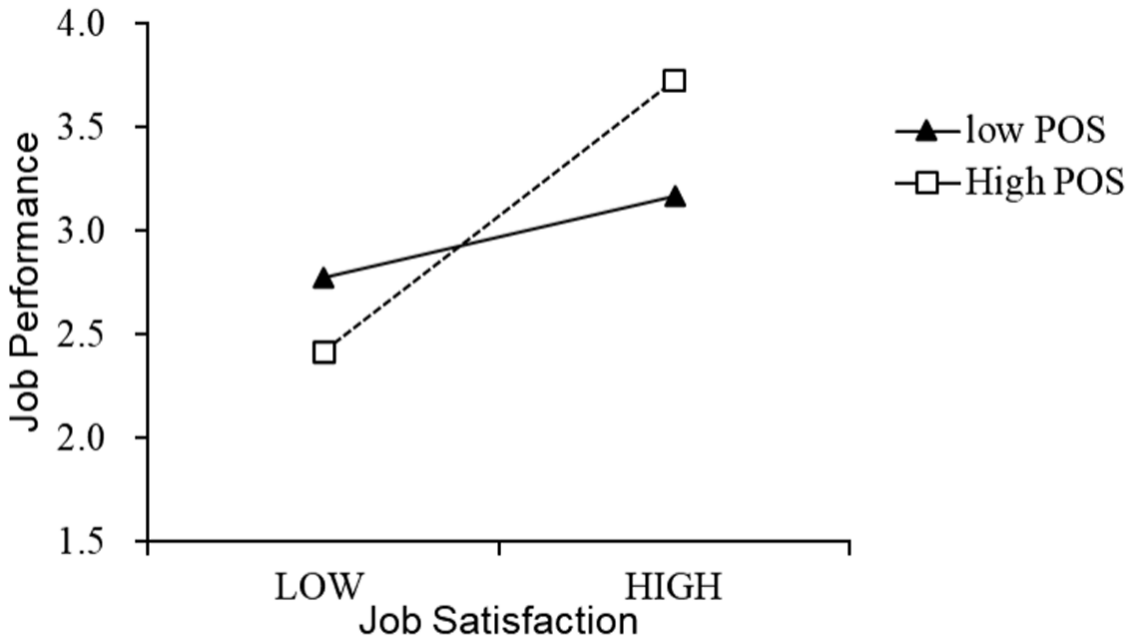
Moderating effect of organizational support.

To show the moderating effect of organizational support more intuitively, this study uses the mean value of the variable plus or minus one standard deviation as the grouping standard to describe the relationship between job satisfaction and job performance under high organizational support and low organizational support levels. The result is shown in Figure 2. The solid line and the dashed line, respectively, indicate the relationship’s strength between employees’ job satisfaction and job performance under low and high levels of organizational support. It can be seen from Figure 2 that the slope of the solid line is lower than the dotted line, which means that for employees with a higher sense of organizational support, employee job satisfaction has a greater degree of positive impact on their work performance. While for employees with a higher sense of organizational support for low-level employees, the degree of the positive impact of employee job satisfaction on their job performance is relatively weak. This evidence shows that organizational support can play a positive moderating role in the relationship between job satisfaction and job performance.

#### 4.4.2 Moderating effect of age discrimination perception

Through regression analysis of job satisfaction, age discrimination perception, and job performance, we can see that the standardized coefficient of job satisfaction * age discrimination perception (that is, the interaction item in Table 8) is −0.849, the *p* value is significant at the level of 0.001; the *F* values of the models are 48.522 and 42.095, respectively;ΔR^2^ is significant at the 0.05 level. These numbers indicate that the regression fits well. In summary, there is a negative moderating effect of age discrimination perception between job satisfaction and job performance; hence, hypothesis H4 is verified.

**Table 8:** Moderating effect analysis of age discrimination perception.

Model	Variable	Non-standardized coefficient		Standardized Coefficient	*t*	Sig.	*F*	*R* ^2^	Adjusted *R*^2^
		*β*	Standard error	*β*					
M10	Constant	1.48	0.266		5.558	0.000			
	JS	0.465	0.051	0.464	9.076	0.000	48.522***	0.196***	0.192***
	ADP	0.047	0.05	0.049	0.952	0.341			
M11	Constant	−0.839	0.542		−1.547	0.123			
	JS	1.241	0.167	0.24	7.428	0.000	42.095***	0.242***	0.236***
	ADP	0.853	0.172	0.877	4.947	0.000			
	JS*ADP	−0.284	0.058	−0.849	−4.868	0.000			

Correlation: *p < 0.05, **p < 0.01, ***p < 0.001.

**Figure 3:**
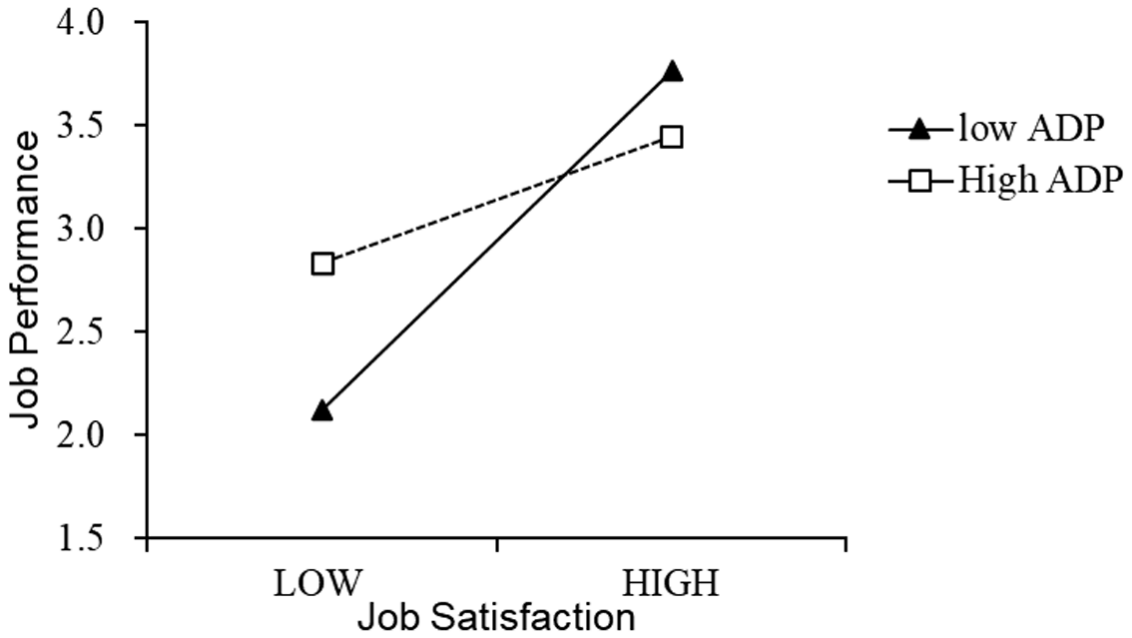
Moderating effect of age discrimination perception.

In the same way, to more intuitively present the moderating effect of age discrimination perception, this study uses the mean value of the variable plus or minus one standard deviation as the grouping standard, respectively, to determine the relationship between job satisfaction and job performance at the high and low levels of age discrimination perception. The relationship is depicted, and the result is shown in Figure 3. The solid line and the dotted line, respectively, indicate the relationship’s strength between employees’ job satisfaction and job performance under low and high levels of age discrimination perception. It can be seen from Figure 3 that the slope of the solid line is higher than the dotted line, which means that for employees with a low perception of age discrimination, employee job satisfaction has a greater degree of positive influence on their work performance. While the perception of age discrimination is more optimistic, for high-level employees, the positive impact of employee job satisfaction on their job performance is relatively weak. This shows that the perception of age discrimination can play a negative moderating role in the relationship between job satisfaction and job performance.

### 4.5 Comparative analysis of job performance between two groups

The hotel industry is a labor-intensive industry, which provides many job opportunities for middle-aged and older adults. Middle-aged and older adults have abundant work experience and life experience to deal with problems, get along with challenges more quickly and effectively, and produce better job performance, which is conducive for enterprises to run efficiently. However, due to the decline in health, middle-aged and elderly employees face different challenges in organizing their work. At the same time, non-middle-aged and elderly employees will also have the corresponding stereotypes of middle-aged and elderly employees, increasing the difficulty of managers. Then based on the human resources management point of view, managers have to realize the differences between middle-aged and elderly employees and non-middle-aged and elderly employees and make a more effective way of staff management.

#### 4.5.1 Comparing the main effect between two groups

According to the previous results, the three-dimensional capital significantly affects the job performance of middle-aged and elderly employees. Therefore, this research aims to analyze the mechanism and essential boundary conditions of three-dimensional capital and job performance of middle-aged and elderly employees. The study is further aimed at analyzing the difference in the effect of three-dimensional capital on job satisfaction between middle-aged and elderly employees and non-middle-aged and elderly employees. Thus, dummy variable D is introduced. When the research subject is middle-aged and elderly employees, D = 0; when the research subject is non-middle-aged and elderly employees, D = 1. To better illustrate the moderating effect of age group, three equations are constructed. Equation (1) presents the interaction model using dummy variable D, while Equations (2, 3) show the specific models for the two subgroups. The model is as follows:


(1) $$Y=\propto_{1}+\propto_{2}D+\beta_{1}X+\beta_{2}\left(D*X\right)+\mu$$

When the subject of the study are middle-aged and elderly employees:


(2) $$Y=\propto_{1}+\beta_{1}X+\mu$$

When the subject of the study are non middle-aged and elderly employees:


(3) $$Y=\left(\propto_{1}+\propto_{2}\right)+\left(\beta_{1}+\beta_{2}\right)X+\mu$$

When $\propto_{2}$ is significant, it represents the two age groups are different in intercept; when $\beta_{2}$ is significant, it represents the two age groups are different in slope.

The effect of the three-dimensional capital on job performance is shown in Table 9. The three-dimensional capital of all employees has a positive effect on job performance (*β* = 0.201, *p* < 0.001; *β* = 0.214, *p* < 0.001; *β* = 0.348, *p* < 0.001). When comparing the results of middle-aged and elderly employees and non-middle-aged and elderly employees, there are significant differences between the effect of human capital, social capital and psychological capital. (*β* = 0.588, *p* < 0.01; *β* = 0.382, *p* < 0.05; *β* = 0.479, *p* < 0.05).

**Table 9:** Results of the effect of three-dimensional capital on job performance between two groups.

Variables	Model 12	Model 13	Model 14
*D*	0.142	0.309	0.245**
Human capital	0.201***		
Human capital *D	0.588***		
Social capital		0.214***	
Social capital *D		0.382**	
Psychological capital			0.348***
Psychological capital *D			0.479**
*R* ^2^	0.526***	0.523***	0.506***
Adjusted *R*^2^	0.523***	0.519***	0.502***
ΔF	146.672***	144.694***	135.041***

Correlation: *p < 0.05, **p < 0.01, ***p < 0.001.

#### 4.5.2 Comparing the mediating effect between two groups

Based on the above analysis, there are differences between middle-aged and elderly employees and non-middle-aged and elderly employees in job performance. From the perspective of the main effects the three-dimensional capital has on job performance, we can learn that although the three-dimensional capital has a particular impact on the employee’s job performance, the most influential factor lies in the middle-aged and elderly employees’ cognition of the job itself, the interpersonal relationship and salary. This study also represents that the mediating effect that the job satisfaction of middle-aged and elderly employees has on three-dimensional capital & job performance is significant but incomplete. Combined with Table 10 below, we can calculate that the ratios of the mediating effect of job satisfaction of middle-aged and elderly employees to the total effect of the three-dimensional capital and job performance are as follows: human capital 0.145, social capital 0.192, and psychological capital 0.247. For non-middle-aged and elderly employees, the ratios of the mediating effect of job satisfaction to the total effect are as follows: human capital 0.466, social capital 0.252, and psychological capital 0.366. The ratios of the mediating effects of middle-aged and elderly employees are, respectively, bigger than that of non-middle-aged and elderly employees, which shows that the mediating effects of job satisfaction in middle-aged and elderly employees are more robust than that of non-middle-aged and elderly employees.

**Table 10:** Results of the mediating effect of job satisfaction between two groups.

Variables	Model 15	Model 16	Model 17
*D*	−0.058	0.073	−0.024
Human capital	0.145***		
Human capital *D	0.466***		
Social capital		0.192***	
Social capital *D		0.252*	
Psychological capital			0.247***
Psychological capital *D			0.366**
Job satisfaction	0.043	0.017	0.043
Job satisfaction *D	0.328**	0.384**	0.391***
*R* ^2^	0.550***	0.547***	0.539***
Adjusted *R*^2^	0.544***	0.541***	0.533***
ΔF	96.308 ***	99.050 ***	92.136 ***

Correlation: *p < 0.05, **p < 0.01, ***p < 0.001.

Further, this study analyzes the differences in the effects of the three dimensions of job satisfaction on job performance between middle-aged and elderly employees and non-middle-aged and elderly employees. As shown in Table 11, for middle-aged and elderly employees, the effect of job satisfaction, interpersonal relationship satisfaction, and salary satisfaction have on job performance is positive and significant (*β* = 0.055, *p* < 0.01; *β* = 0.012, *p* < 0.001; *β* = 0.049, *p* < 0.001). For non-middle-aged and elderly employees, the effect of job satisfaction and salary satisfaction on job performance is significantly weaker than that of middle-aged and elderly employees, and the gap is significant (*β* = 0.428, *p* < 0.001; *β* = 0.465, *p* < 0.001;*β* = 0.467, *p* < 0.001).

**Table 11:** Results of the mediating effect of the three dimensions of job satisfaction between two groups.

Variables	Model 18	Model 19	Model 20
*D*	0.276**	0.257*	0.241**
Job	0.055**		
Job *D	0.428***		
Interpersonal relationship		0.012***	
Interpersonal relationship *D		0.465***	
Salary			0.049***
Salary *D			0.467***
*R* ^2^	0.519***	0.510**	0.521***
Adjusted *R*^2^	0.515**	0.506***	0.517**
ΔF	142.296 ***	137.269***	143.628***

Correlation: *p < 0.05,**p < 0.01, ^***^p < 0.001.

#### 4.5.3 Comparing the moderating effect between two groups

The previous data analysis shows that perceived organizational support has a moderating effect on job satisfaction and job performance. However, the difference between the perceived organizational support of middle-aged and elderly employees (*β* = −0.083, *p* > 0.05) and the perceived organizational support of non-middle-aged and elderly employees is not significant (Table 12).

**Table 12:** Results of the moderating effect of the perceived organizational support between two groups.

Variables	Model 21	Model 22
D	0.237*	0.359**
Job satisfaction	0.049	−0.368**
Job satisfaction *D	0.589***	0.680***
Perceived organizational support	−0.020	−0.388**
Perceived organizational support *D	−0.121	−0.292*
JS*POS		0.736**
JS*POS *D		−0.083
*R* ^2^	0.524***	0.533***
Adjusted *R*^2^	0.518***	0.524
ΔF	86.750***	63.797***

JS = Job Satisfaction; POS = Perceived Organizational Support. Correlation: *p < 0.05, **p < 0.01, ^***^p < 0.001.

According to the data analysis, we have learned that age discrimination perception plays a moderator in job satisfaction and job performance. Table 13 shows that the perception of age discrimination among middle-aged and elderly employees has both a significant and negative effect on the reconciliation between job satisfaction and job performance. Compared to that of middle-aged and elderly employees, the moderating effect gap of age discrimination perception in non-middle-aged and elderly employees between job satisfaction and job performance is not significant (*β* = −0.685, *p* > 0.05).

**Table 13:** Results of the moderating effect of the age discrimination perception between two groups.

Variables	Model 23	Model 24
*D*	0.032	−0.713
Job satisfaction	0.040	0.342
Job satisfaction*D	0.579***	1.035**
Age discrimination perception	−0.006	0.284
Age discrimination perception*D	0.122	0.866*
JS*ADP		−0.326
JS*ADP*D		−0.685
*R* ^2^	0.523***	0.541***
Adjusted *R*^2^	0.517***	0.533***
ΔF	86.238 ^***^	66.104^***^

JS = Job Satisfaction; AD = age discrimination perception. Correlation: *p < 0.05, **p < 0.01, ***p < 0.001.

## 5 Conclusion

### 5.1 Discussion of results

This research focuses on four questions. Does the three-dimensional capital of middle-aged and elderly employees affect their job performance? Does job satisfaction mediate the three-dimensional capital of middle-aged and elderly employees and job performance? Do perceived organizational support and age discrimination perceptions moderate the job satisfaction and job performance of middle-aged and elderly employees? Is there any difference between the three-dimensional capital’s primary effects, the mediating effect of job satisfaction, the moderating effect of perceived organizational support, and age discrimination perception between middle-aged and elderly employees and non-middle-aged and elderly employees? The following points are summaries of the results of this study.

First, this research analyzes the impact of the three significant capitals of hotel employees on job performance. The research results show that the three major capitals of middle-aged and elderly employees in the hotel industry significantly impact their work performance. From the perspective of group comparison, the human capital, social capital and psychological capital of non-middle-aged and elderly employees has significant impact on work performance. Based on the sample situation of this survey, there are more non-middle-aged and elderly employees in the hotel industry. Compared with middle-aged and older adults, young and middle-aged people have less social experience and lack resources in the same industry or other industries. Due to excessive survival pressure, their psychological resilience may also generally be worse. Similar to the findings of Huang et al., the three-dimensional capital of middle-aged and elderly employees has significant impact on work performance (Huang et al., 2021). In general, the three-dimensional capital of employees is the key factor affecting employee behavior (Li et al., 2021). Business managers should formulate relevant training policies for employees’ human capital, social capital, and psychological capital to stimulate employees’ work performance and improve the organization’s competitiveness.

Second, the test of the mediator role of job satisfaction shows that the employee’s job satisfaction plays a partial mediator role in the employee’s three-dimensional capital and job performance. This finding is consistent with Haque and Oino’s (2019) studies. According to the research sample, since the job responsibilities of each department in the hotel and the job content of each position are relatively straightforward, there are few conflicts of interest between employees, so the interpersonal atmosphere at work is relatively straightforward. Therefore, interpersonal relationship satisfaction has less impact on job performance.

Third, through the test of the moderating effect of employees’ sense of organizational support, it is shown that the sense of organizational support will positively and significantly regulate the impact of job satisfaction on job performance. This finding enriches the studies on organizational support (Wen et al., 2019). The possible reason is that compared with employees with high organizational support, employees with low organizational support are more sensitive to job satisfaction. As a result, when the organization’s sense of support increases to a certain extent, employees’ job performance will be significantly improved. This conclusion shows that organizational support is an important variable that affects the relationship between hotel staff’s job satisfaction and job performance and should be paid attention to.

Fourth, the test on the moderating effect of employees’ perception of age discrimination shows that perception of age discrimination negatively regulates the impact of job satisfaction on job performance. Specifically, the perceived job satisfaction of employees who merely feel age discrimination has a more significant impact on job performance. From the perspective of group comparison, the age discrimination perception of middle-aged and old employees has a significant negative adjustment to work performance. In contrast, the age discrimination perception of non-middle-aged and old employees has no significant effect on the adjustment of work performance. This is because middle-aged and elderly employees are more likely to be stereotyped as having low work efficiency because of their age and are more restricted in their work (Neumark, 2018). Therefore, to improve the work performance of middle-aged and elderly employees, in addition to improving employees’ job satisfaction, we must also pay attention to weakening employees’ perception of age discrimination.

### 5.2 Theoretical implication

There are three contributions to this study. First, it enriches the research of middle-aged and elderly employees. The current research about the middle-aged and elderly groups focuses on qualitative research, and quantitative research is relatively limited; studies in the hotel industry are even more limited. Our findings enriched the existing research foundation and angle of middle-aged and elderly employees by collecting questionnaire data, studying the correlation between three-dimensional capital and job performance quantitatively, and introducing dummy variables in models to analyze the differences between middle-aged and elderly employees and non-middle-aged and elderly employees.

Second, it discusses the synergy of the three-dimensional capital on job satisfaction and job performance. This research found that the synergy of the three-dimensional capital can significantly affect job satisfaction and job performance, which shows that employers should pay attention to improving a specific capital and pay attention to the synergy and substitution of the human capital, social capital, and psychological capital. To some extent, this study also complements the limitations of the current study on the three capitals’ impacts on job performance.

Third, it explains the moderator role of age discrimination perception in job satisfaction and job performance. Especially for middle-aged and elderly employees, the stronger their perception of age discrimination, the lower their job performance. However, the moderating effect of age discrimination perceptions of non-middle-aged and elderly employees is not significant. This provides the corresponding theoretical basis for the organization to formulate different management policies for different age groups.

### 5.3 Practical implications

Global aging is inevitable, as the hotel industry is a labor-intensive industry with a lower employment threshold, there will be more and more middle-aged and elderly workers joining in. While this study is based on data from Taiwan’s hospitality industry, its findings have global relevance. The role of three-dimensional capital in improving job performance is not limited to the hospitality sector but can be applied to other industries with aging workforces, such as healthcare, education, and customer service. Similarly, the findings on age discrimination and its impact on job performance can inform organizational policies worldwide, helping businesses create more inclusive environments for workers of all ages. Given the four questions raised at the beginning of this study, the study provides the following relevant human resources management recommendations.

#### 5.3.1 Build a positive corporate culture

A positive organizational culture is a basis for the collaborative development of employees’ human capital, social capital, and psychological capital. Thus, it is necessary to replace negative punishments and restrictions with a positive incentive system and enrich employees’ work tasks through the horizontal expansion of the scope of work. First, building a project-based work system can allow employees to participate in the work of different departments. The second is to establish a rotation system to allow employees to sign up or select outstanding employees to work in other departments for a period to be exposed to different work tasks. Flexible work arrangements could also be introduced to help older employees better balance work and life, thereby improving their job performance.

#### 5.3.2 Improve the hotel’s IQ, EQ, and the adverse quotient

Under the high-intensity social work rhythm, work pressure and its impact on employees’ personal family life are increasing, and practical guidance is particularly critical. Collaborative cultivation of employees’ IQ, EQ, and adverse quotient is essential for improving employees’ comprehensive ability and quality. In addition to providing traditional vocational skills and job skills training to enrich employees’ human capital, the human resources department should also provide more opportunities for communication to increase employees’ social capital. Additionally, organizations should develop dedicated mental health support programs to enhance the psychological capital of middle-aged and elderly workers, helping them cope with work challenges and boosting their self-efficacy and job satisfaction.

#### 5.3.3 Improve middle-aged and elderly employees’ job satisfaction

Job satisfaction will directly affect employees’ job performance. Employee job satisfaction is divided into three dimensions, job satisfaction, interpersonal relationship satisfaction, and salary satisfaction, so there are three ways to improve employee job satisfaction. First, improve the job itself. Recruit employees who are interested in the job, adjust the job content appropriately to increase the challenge and attractiveness of the job, carry out job rotation to help employees find their dream positions. Second, improve the interpersonal relationship. Employees’ interpersonal relationship is accomplished through team building, from building formal and informal teams, respectively. Third, improve salary satisfaction. Effective communication to understand the needs of employees is necessary for formulating a salary management system that is attractive to employees. While job satisfaction partially mediates this relationship, the research suggests that certain job aspects, like job content and interpersonal relationships, need to be enhanced. Organizations should focus on improving job roles by increasing variety, responsibility, and meaningfulness, as well as ensuring that employees have opportunities for career advancement and personal growth.

#### 5.3.4 Improve middle-aged and elderly employees’ perceived organizational support

Perceived organizational support is divided into work support and social–emotional support. Hence, hotel managers should pay attention to developing a scientific management system to ensure the welfare of employees easy access to work resources, and smooth feedback of problems. Besides, it is also necessary to take good care of employees’ emotions, to help them solve work doubts and handle negative emotions. So that employees could build a strong sense of trust with the organization.

#### 5.3.5 Weaken middle-aged and elderly employees’ age discrimination perception within the organization

Middle-aged and elderly employees want to be treated equally in their work, not discriminated against by their age. The hotels should formulate more scientific and objective regulations on human resource management and employee assessment to avoid age-based stereotypes that limit middle-aged and elderly employees’ work, hurt their motivation, and finally negatively affect job performance. With the aging population, middle-aged and elderly employees will be an essential source of human resources for the hotel industry, and this deserves much attention from hotel employers and managers.

### 5.4 Limitations and future research

This study collects data from 400 hotel employees and their supervisors through a questionnaire to study the influence mechanism of the three-dimensional capital on job performance. However, due to the research methods and sample size, there are still some limitations. And with the continuous expansion of the population aging, it is imperative to study the work environment of middle-aged and elderly employees the factors affecting their job performance, and the influence mechanism. In future studies, we still have to make efforts in the following areas.

1. To further refine the influence that three-dimensional capital has on middle-aged and elderly employees’ job performance. This study puts forward that three-dimensional capital are essential factor affecting middle-aged and elderly employees’ job performance and discusses the synergy among three capitals. It does not elaborate on the impact of specific dimensions of each capital on job performance. Therefore, the effect of each dimension of three-dimensional capital on job performance should be tested in future studies.
2. To study other factors’ impacts on middle-aged and elderly employees on job performance. This study mainly discusses the influence of three-dimensional capital on job performance and the mediating effect of job satisfaction. In addition, this study chooses perceived organizational support and age discrimination perception as two critical moderators. However, other situational factors, such as corporate culture and leadership style, also need to be considered when measuring employees’ job performance. Therefore, a higher level of organizational and team scenarios could be considered in future studies.
3. This study measures the employees’ job performance by sending questionnaires to their supervisors. There are inevitably errors in its authenticity and accuracy since this approach only reflects supervisors’ evaluation of employees’ job performance. Future studies require more effective measurement of job performance, such as employee self-evaluate combined with supervisor evaluation.
4. This study employed a convenience sampling method, primarily due to considerations of time, cost, and resource feasibility. While this approach was practical for the current study, future research could benefit from employing more representative sampling methods, such as stratified or random sampling, to further enhance the external validity and generalizability of the findings.

## Data Availability

The original contributions presented in the study are included in the article/Supplementary material, further inquiries can be directed to the corresponding author/s.
