# Supplementary material for: Work performance of middle-aged and elderly employees in hotel industry: the moderating effects of organizational support and age discrimination
Source: Front Psychol. 2025 Apr 28;16:1377368. doi: 10.3389/fpsyg.2025.1377368 (PMC12038056; doi:10.3389/fpsyg.2025.1377368)
Supplement: Supplementary file 1 [file Table_1.docx]

# Appendix A

**Questionnaire on Three Dimensional Capital and Job Satisfaction of Hotel Industry Employees**

Hello! This questionnaire is aimed at investigating the three-dimensional capital, work performance, and job satisfaction of hotel employees. Please take a few minutes to fill out this questionnaire carefully. Thank you very much for your help!

Department of Tourism and Hotel Management, School of Management, Xiamen University

**Part 1 Basic Situation Investigation**

1. Your age____

2. Your gender____

A. Male B Female

3. Your level of education

A. High school and below

B Associate degree

C Undergraduate

D Master's degree or above

4. Your hotel is

A. Three star hotels and below

B Four star hotels

C. Five star hotels

5. Your employee number___________

**Part 2 Investigation of 3D Capital Situation**

Please rate each item from 1 to 5, indicating strongly disagree to strongly agree. Please make a rating based on the actual situation.

| Items | Strongly disagree -->Strongly agree | | | | |
| --- | --- | --- | --- | --- | --- |
| 1. Compared to my colleagues, my professional theoretical knowledge is more extensive | 1 | 2 | 3 | 4 | 5 |
| 2. Compared to my colleagues, I have more work experience | 1 | 2 | 3 | 4 | 5 |
| 3. Compared to my colleagues, my professional skills are higher | 1 | 2 | 3 | 4 | 5 |
| 4. Compared to my colleagues, my physical condition is healthier | 1 | 2 | 3 | 4 | 5 |
| 5. Among my colleagues, I have a lot of good friends | 1 | 2 | 3 | 4 | 5 |
| 6. In leadership, they all trust me very much | 1 | 2 | 3 | 4 | 5 |
| 7. Outside the department, I also have many friends in the industry | 1 | 2 | 3 | 4 | 5 |
| 8. I am able to analyze long-term problems and find solutions | 1 | 2 | 3 | 4 | 5 |
| When participating in management meetings, I am confident in introducing things within my scope of work | 1 | 2 | 3 | 4 | 5 |
| 10. I contribute to the company's strategy | 1 | 2 | 3 | 4 | 5 |
| 11. I can help set goals | 1 | 2 | 3 | 4 | 5 |
| 12. I can discuss with people outside the company | 1 | 2 | 3 | 4 | 5 |
| 13. I am able to present information to colleagues | 1 | 2 | 3 | 4 | 5 |
| 14. If I encounter difficulties in my work, I will come up with solutions | 1 | 2 | 3 | 4 | 5 |
| Currently, I always have a full spirit when completing work | 1 | 2 | 3 | 4 | 5 |
| No matter what problem, there are always many solutions | 1 | 2 | 3 | 4 | 5 |
| 17. I think my current job is very outstanding | 1 | 2 | 3 | 4 | 5 |
| 18. Faced with current work goals, I can come up with many methods to achieve them | 1 | 2 | 3 | 4 | 5 |
| Currently, I have set work goals for myself and am working hard to achieve them | 1 | 2 | 3 | 4 | 5 |
| 20. If there is a setback in my work, I will step out and continue working in a short period | 1 | 2 | 3 | 4 | 5 |
| 21. When encountering difficulties at work, I will try my best to solve them | 1 | 2 | 3 | 4 | 5 |
| 22. If I encounter work that I have to do, I can also complete it independently | 1 | 2 | 3 | 4 | 5 |
| 23. Faced with work pressure, my mentality is very calm | 1 | 2 | 3 | 4 | 5 |
| 24. I have encountered too many hardships in the past, so for me, the difficulties in work are only temporary | 1 | 2 | 3 | 4 | 5 |
| 25. In my current job, I can handle many things simultaneously | 1 | 2 | 3 | 4 | 5 |
| 26. When there is uncertainty in my work, I am confident in the best outcome | 1 | 2 | 3 | 4 | 5 |
| 27. Disadvantages that arise in the workplace are always short-lived and solutions will always be found | 1 | 2 | 3 | 4 | 5 |
| 28. What I see in my work is bright content | 1 | 2 | 3 | 4 | 5 |
| 29. I hold an optimistic attitude towards the uncertainty of my future work | 1 | 2 | 3 | 4 | 5 |
| 30. Current work related matters are developing as expected | 1 | 2 | 3 | 4 | 5 |

**Part 3 Job Satisfaction Survey**

| Items | Strongly disagree -->Strongly agree | | | | |
| --- | --- | --- | --- | --- | --- |
| 32. I am satisfied with the nature of the work I am undertaking | 1 | 2 | 3 | 4 | 5 |
| 33. I am satisfied with the results I have obtained from my work | 1 | 2 | 3 | 4 | 5 |
| I am satisfied with the opportunities to present my own ideas and take action in my work | 1 | 2 | 3 | 4 | 5 |
| 35. I am satisfied with the challenges in my work | 1 | 2 | 3 | 4 | 5 |
| 36. I am satisfied with the people I talk to and work with | 1 | 2 | 3 | 4 | 5 |
| 37. I am satisfied with the respect and fair treatment I have received from my superiors | 1 | 2 | 3 | 4 | 5 |
| 38. I am satisfied with the opportunity to interact with others during work | 1 | 2 | 3 | 4 | 5 |
| 39. I am satisfied with the support and guidance I received from my superiors | 1 | 2 | 3 | 4 | 5 |
| 40. I am satisfied with the salary and various benefits I have received | 1 | 2 | 3 | 4 | 5 |
| I am satisfied with the fairness of personnel promotion in my organization | 1 | 2 | 3 | 4 | 5 |
| 42. I am satisfied with the consistency between the compensation received and the contributions I have made to the organization | 1 | 2 | 3 | 4 | 5 |
| 43. I am satisfied with my promotion opportunities at work | 1 | 2 | 3 | 4 | 5 |

**Part 4 Investigation of Organizational Support**

| Items | Strongly disagree -->Strongly agree | | | | |
| --- | --- | --- | --- | --- | --- |
| 44. The organization values my opinions | 1 | 2 | 3 | 4 | 5 |
| 45. The organization cares about my welfare | 1 | 2 | 3 | 4 | 5 |
| 46. The organization values my goals and values | 1 | 2 | 3 | 4 | 5 |
| 47. When I encounter difficulties, the organization will help me | 1 | 2 | 3 | 4 | 5 |
| 48. The organization will forgive my unintentional mistakes | 1 | 2 | 3 | 4 | 5 |
| 49. The organization has assigned me the most suitable job for me | 1 | 2 | 3 | 4 | 5 |
| 50. Organizations rarely care about me | 1 | 2 | 3 | 4 | 5 |
| 51. If I need special assistance, the organization is willing to help me | 1 | 2 | 3 | 4 | 5 |

**Part 5 Investigation on Age Discrimination**

| Items | Strongly disagree -->Strongly agree | | | | |
| --- | --- | --- | --- | --- | --- |
| 52. I have been fired before due to age reasons | 1 | 2 | 3 | 4 | 5 |
| 53. Due to my age, my contribution has become less valued | 1 | 2 | 3 | 4 | 5 |
| 54. Due to my age, the opportunities for me to express my ideas are decreasing | 1 | 2 | 3 | 4 | 5 |
| 55. Due to my age, I have received unfair evaluations before | 1 | 2 | 3 | 4 | 5 |
| 56. Due to my age, I have received less social support | 1 | 2 | 3 | 4 | 5 |
| 57. Due to my age, people have always believed that my abilities are weak | 1 | 2 | 3 | 4 | 5 |
| 58. Due to my age, I receive less respect | 1 | 2 | 3 | 4 | 5 |
| 59. Due to my age, someone has delayed or ignored my request | 1 | 2 | 3 | 4 | 5 |
| 60. Someone accused me of failing or having problems due to my age | 1 | 2 | 3 | 4 | 5 |

Finally, thank you for your help and cooperation.

Wishing you all the best in your work!

# Appendix B.

**Questionnaire for measuring work performance of hotel employees**

Hello! This questionnaire is a survey on the work performance of hotel employees. Please take a few minutes to carefully fill out this questionnaire and evaluate the work performance of your subordinate employees. Thank you very much for your help!

**Employee ID:__________________**

Please rate each item from 1 to 5, indicating strongly disagree to strongly agree. Please make a rating based on the actual situation.

| Items | Strongly disagree -->Strongly agree | | | | |
| --- | --- | --- | --- | --- | --- |
| 1. The employee's workload is higher than the average level | 1 | 2 | 3 | 4 | 5 |
| 2. The employee's work quality is much higher than the average level | 1 | 2 | 3 | 4 | 5 |
| 3. The employee's work efficiency is much higher than the average level | 1 | 2 | 3 | 4 | 5 |
| 4. The employee's work quality standards are higher than the general standards for the job | 1 | 2 | 3 | 4 | 5 |
| 5. The employee strives to pursue work of higher quality than required | 1 | 2 | 3 | 4 | 5 |
| 6. The employee adheres to the highest professional standards | 1 | 2 | 3 | 4 | 5 |
| 7. The employee's ability to perform core work tasks | 1 | 2 | 3 | 4 | 5 |
| 8. The employee's judgment ability in executing core work tasks | 1 | 2 | 3 | 4 | 5 |
| 9. The accuracy of the employee in executing core work tasks | 1 | 2 | 3 | 4 | 5 |
| 10. The employee's work knowledge about core work tasks | 1 | 2 | 3 | 4 | 5 |
| 11. The employee's creativity in executing core tasks | 1 | 2 | 3 | 4 | 5 |

Finally, thank you for your help and cooperation. Wishing you all the best in your work!
