# Supplementary material for: Work performance of middle-aged and elderly employees in hotel industry: the moderating effects of organizational support and age discrimination
Source: Front Psychol. 2025 Apr 28;16:1377368. doi: 10.3389/fpsyg.2025.1377368 (PMC12038056; doi:10.3389/fpsyg.2025.1377368)
Supplement: Supplementary file 2 [file Table_2.docx]

# 附录A

**酒店业员工三维资本与工作满意度测评问卷**

您好！本问卷是针对酒店员工三维资本、工作绩效、工作满意度的调查，恳请您花几分钟时间认真填写这份问卷，非常感谢您的帮助！

厦门大学管理学院旅游与酒店管理系

**第一部分 基本情况调查**

| 1.您的年龄____ | | | | |
| --- | --- | --- | --- | --- |
| 2.您的性别____ | | | | |
| A. 男 | B. 女 |  |  |  |
| 3.您的受教育程度 | | | | |
| A. 高中及以下 | B. 大专 | C. 本科 | D. 硕士及以上 |  |
| 4. 您所在的酒店为 | | | | |
| A. 三星级酒店及以下 | B. 四星级酒店 | C.五星级酒店 |  |  |
| 5. 您的员工编号___________ | | | | |

**第二部分 三维资本情况调查**

请您针对各项目进行评分，由1到5分别表示非常不同意到非常同意，请根据实际情况做出评价。

| 题目 | 非常不同意---->非常同意 | | | | |
| --- | --- | --- | --- | --- | --- |
| 1. 与同事相比，我的专业理论知识更丰富 | 1 | 2 | 3 | 4 | 5 |
| 2. 与同事相比，我的工作经验更丰富 | 1 | 2 | 3 | 4 | 5 |
| 3. 与同事相比，我的专业技能水平更高 | 1 | 2 | 3 | 4 | 5 |
| 4. 与同事相比，我的身体情况更健康 | 1 | 2 | 3 | 4 | 5 |
| 5. 在同事中，我的好朋友非常多 | 1 | 2 | 3 | 4 | 5 |
| 6. 在领导中，他们都对我很信任 | 1 | 2 | 3 | 4 | 5 |
| 7. 在部门外，我也有很多业内的好友 | 1 | 2 | 3 | 4 | 5 |
| 8. 我能分析长远的问题并找到对策 | 1 | 2 | 3 | 4 | 5 |
| 9. 参与管理层会议的时候，对于介绍自己工作范围内的事情，我很有信心 | 1 | 2 | 3 | 4 | 5 |
| 10.我对公司战略有贡献 | 1 | 2 | 3 | 4 | 5 |
| 11. 我能帮助设定目标 | 1 | 2 | 3 | 4 | 5 |
| 12. 我能与公司外部的人讨论 | 1 | 2 | 3 | 4 | 5 |
| 13. 我能向同事陈述信息 | 1 | 2 | 3 | 4 | 5 |
| 14. 倘若我在工作中遭遇了难题，我会想出解决办法 | 1 | 2 | 3 | 4 | 5 |
| 15. 目前，我在完成工作的时候总是有着饱满的精神 | 1 | 2 | 3 | 4 | 5 |
| 16.不管什么问题，解决方法总是很多的 | 1 | 2 | 3 | 4 | 5 |
| 17. 我觉得当下自己的工作是很出色的 | 1 | 2 | 3 | 4 | 5 |
| 18. 面对当下的工作目标，我可以想出很多方法来完成 | 1 | 2 | 3 | 4 | 5 |
| 19. 目前，我已经为自己设置了工作目标，并且正在努力完成 | 1 | 2 | 3 | 4 | 5 |
| 20. 工作出现了挫折，我会在短时间内走出来，继续工作 | 1 | 2 | 3 | 4 | 5 |
| 21. 工作中遇到了难题，我会想方设法的解决好 | 1 | 2 | 3 | 4 | 5 |
| 22. 如果遇到不得不做的工作，我也可以独立完成 | 1 | 2 | 3 | 4 | 5 |
| 23. 面对工作压力，我的心态很平和 | 1 | 2 | 3 | 4 | 5 |
| 24. 从前遭遇过的磨难太多了，所以对我而言，工作上的困难只是暂时的 | 1 | 2 | 3 | 4 | 5 |
| 25. 在我目前的工作中，我可以同时处理很多事情 | 1 | 2 | 3 | 4 | 5 |
| 26. 工作中如果出现了不确定的事情时，我会对最好的结果充满信心 | 1 | 2 | 3 | 4 | 5 |
| 27. 工作中出现的不利事情，总是短暂的，总会找到办法解决 | 1 | 2 | 3 | 4 | 5 |
| 28. 对自己的工作，我看的都是光明的内容 | 1 | 2 | 3 | 4 | 5 |
| 29. 我对工作未来的不确定持乐观心态 | 1 | 2 | 3 | 4 | 5 |
| 30. 当前与工作有关的事情正在如预期那样发展 | 1 | 2 | 3 | 4 | 5 |

**第三部分 工作满意度情况调查**

| 题目 | 非常不同意---->非常同意 | | | | |
| --- | --- | --- | --- | --- | --- |
| 32. 我对所担任工作的性质感到满意 | 1 | 2 | 3 | 4 | 5 |
| 33. 我对从工作中获得的成绩感感到满意 | 1 | 2 | 3 | 4 | 5 |
| 34. 我对工作中可以提出自己想法和付诸行动的机会感到满意 | 1 | 2 | 3 | 4 | 5 |
| 35. 我对工作中的挑战性感到满意 | 1 | 2 | 3 | 4 | 5 |
| 36. 我对与我交谈和共事的人感到满意 | 1 | 2 | 3 | 4 | 5 |
| 37. 我对从上级那里得到的尊重和受到的公平待遇感到满意 | 1 | 2 | 3 | 4 | 5 |
| 38. 我对工作的时候能够与他人交往的机会感到满意 | 1 | 2 | 3 | 4 | 5 |
| 39. 我对从上级那里得到的支持和指导感到满意 | 1 | 2 | 3 | 4 | 5 |
| 40. 我对所得的薪酬和各种福利感到满意 | 1 | 2 | 3 | 4 | 5 |
| 41. 我对所在组织中人员晋升的公平性感到满意 | 1 | 2 | 3 | 4 | 5 |
| 42. 我对所得报酬与我为组织所做贡献的一致程度感到满意 | 1 | 2 | 3 | 4 | 5 |
| 43. 我对我在工作中的晋升机会感到满意 | 1 | 2 | 3 | 4 | 5 |

**第四部分 组织支持情况调查**

| 题目 | 非常不同意---->非常同意 | | | | |
| --- | --- | --- | --- | --- | --- |
| 44. 组织重视我的意见 | 1 | 2 | 3 | 4 | 5 |
| 45. 组织关心我的福利 | 1 | 2 | 3 | 4 | 5 |
| 46. 组织重视我的目标和价值 | 1 | 2 | 3 | 4 | 5 |
| 47. 当我遇到困难时，组织会帮助我 | 1 | 2 | 3 | 4 | 5 |
| 48. 组织会原谅我的无心之过 | 1 | 2 | 3 | 4 | 5 |
| 49. 组织让我承担最适合我的工作 | 1 | 2 | 3 | 4 | 5 |
| 50. 组织很少关心我 | 1 | 2 | 3 | 4 | 5 |
| 51. 如果我需要特殊的帮助，组织愿意帮助我 | 1 | 2 | 3 | 4 | 5 |

**第五部分 年龄歧视情况调查**

| 题目 | 非常不同意--->非常同意 | | | | |
| --- | --- | --- | --- | --- | --- |
| 52.我曾由于年龄原因被解雇过 | 1 | 2 | 3 | 4 | 5 |
| 53.由于我的年龄，我的贡献变得不被重视了 | 1 | 2 | 3 | 4 | 5 |
| 54.由于年龄的原因，我表达想法的机会越来越少 | 1 | 2 | 3 | 4 | 5 |
| 55.由于我的年龄，我曾收到过不公正的评价 | 1 | 2 | 3 | 4 | 5 |
| 56.由于年龄的原因，我得到的社会支持较少 | 1 | 2 | 3 | 4 | 5 |
| 57.由于年龄的原因，人们一直认为我能力较弱 | 1 | 2 | 3 | 4 | 5 |
| 58.由于年龄的原因，我受到的尊重较少 | 1 | 2 | 3 | 4 | 5 |
| 59.由于我的年龄，有人拖延或忽视了我的请求 | 1 | 2 | 3 | 4 | 5 |
| 60.有人指责我因年龄原因而失败或出现问题 | 1 | 2 | 3 | 4 | 5 |

最后，谢谢您的帮助和配合，祝您工作顺利！

# 附录B.

**酒店员工工作绩效测量问卷**

您好！本问卷是针对酒店员工工作绩效的调查，恳请您花几分钟时间认真填写这份问卷，对您的下属员工工作绩效进行评价，非常感谢您的帮助！

**员工编号:__________________**

请您针对各项目进行评分，由1到5分别表示非常不同意到非常同意，请根据实际情况做出评价。

| 题目 | 非常不同意--->非常同意 | | | | |
| --- | --- | --- | --- | --- | --- |
| 1.该员工的工作量高于平均水平 | 1 | 2 | 3 | 4 | 5 |
| 2.该员工的工作质量远高于平均水平 | 1 | 2 | 3 | 4 | 5 |
| 3.该员工的工作效率远高于平均水平 | 1 | 2 | 3 | 4 | 5 |
| 4.该员工的工作质量标准高于该工作的通用标准 | 1 | 2 | 3 | 4 | 5 |
| 5.该员工努力追求比要求更高质量的工作 | 1 | 2 | 3 | 4 | 5 |
| 6.该员工坚持最高的职业标准 | 1 | 2 | 3 | 4 | 5 |
| 7.该员工执行核心工作任务的能力 | 1 | 2 | 3 | 4 | 5 |
| 8.该员工在执行核心工作任务时的判断能力 | 1 | 2 | 3 | 4 | 5 |
| 9.该员工在执行核心工作任务时的准确性 | 1 | 2 | 3 | 4 | 5 |
| 10.该员工关于核心工作任务的工作知识 | 1 | 2 | 3 | 4 | 5 |
| 11.该员工在执行核心任务时的创造力 | 1 | 2 | 3 | 4 | 5 |

最后，谢谢您的帮助和配合，祝您工作顺利！
